# Recurrent preterm birth: data from the study “Birth in Brazil”

**DOI:** 10.11606/s1518-8787.2022056003527

**Published:** 2022-03-04

**Authors:** Barbara Almeida Soares Dias, Maria do Carmo Leal, Katrini Guidolini Martinelli, Marcos Nakamura-Pereira, Ana Paula Esteves-Pereira, Edson Theodoro dos Santos

**Affiliations:** I Fundação Oswaldo Cruz Escola Nacional de Saúde Pública Pós-Graduação em Epidemiologia em Saúde Pública Rio de Janeiro RJ Brasil Fundação Oswaldo Cruz. Escola Nacional de Saúde Pública. Pós-Graduação em Epidemiologia em Saúde Pública. Rio de Janeiro, RJ, Brasil; II Fundação Oswaldo Cruz Escola Nacional de Saúde Pública Departamento de Epidemiologia e Métodos Quantitativos em Saúde Rio de Janeiro RJ Brasil Fundação Oswaldo Cruz. Escola Nacional de Saúde Pública. Departamento de Epidemiologia e Métodos Quantitativos em Saúde. Rio de Janeiro, RJ, Brasil; III Universidade Federal do Espírito Santo Programa de Pós-Graduação em Saúde Coletiva Vitória ES Brasil Universidade Federal do Espírito Santo. Programa de Pós-Graduação em Saúde Coletiva. Vitória, ES, Brasil; IV Fundação Oswaldo Cruz Instituto Fernandes Figueira Rio de Janeiro RJ Brasil Fundação Oswaldo Cruz. Instituto Fernandes Figueira. Rio de Janeiro, RJ, Brasil; V Universidade Federal do Espírito Santo Departamento de Medicina Social Vitória ES Brasil Universidade Federal do Espírito Santo. Departamento de Medicina Social. Vitória, ES, Brasil

**Keywords:** Premature Birth, epidemiology, Risk Factors, Propensity Score, Reproductive History, Health Surveys

## Abstract

**OBJECTIVE:**

Describe and estimate the rate of recurrent preterm birth in Brazil according to the type of delivery, weighted by associated factors.

**METHODS:**

We obtained data from the national hospital-based study “Birth in Brazil”, conducted in 2011 and 2012, from interviews with 23,894 women. Initially, we used the chi-square test to verify the differences between newborns according to previous prematurity and type of recurrent prematurity. Sequentially, we applied the propensity score method to balance the groups according to the following covariates: maternal age, socio-economic status, smoking during pregnancy, parity, previous cesarean section, previous stillbirth or neonatal death, chronic hypertension and chronic diabetes. Finally, we performed multiple logistic regression to estimate the recorrence.

**RESULTS:**

We analyzed 6,701 newborns. The rate of recurrence was 42.0%, considering all women with previous prematurity. Among the recurrent premature births, 62.2% were spontaneous and 37.8% were provider-initiated. After weighting by propensity score, we found that women with prematurity have 3.89 times the chance of having spontaneous recurrent preterm birth (ORaj = 3.89; 95%CI 3.01–5.03) and 3.47 times the chance of having provider-initiated recurrent preterm birth (ORaj = 3.47; 95%CI 2.59–4.66), compared to women who had full-term newborns.

**CONCLUSIONS:**

Previous prematurity showed to be a strong predictor for its recurrence. Thus, expanding and improving the monitoring and management of pregnant women who had occurrence of prematurity strongly influence the reduction of rates and, consequently, the reduction of infant morbidity and mortality risks in the country.

## INTRODUCTION

Recurrent prematurity happens when two or more deliveries occur before 37 weeks of gestation^1^ . Although its etiology is complex, multifactorial and even unknown, the scientific literature shows that the occurrence of prematurity comprises one of the main factors for its incidence in subsequent pregnancies^1^ .

The rate of prematurity has increased worldwide, mainly due to the increase in late prematurity, often associated with obstetric interventions^5^ . In 2014, the global rate of prematurity was 10.6 per 100 live births, with Asia accounting for 52.9% of these births. Brazil ranks ninth among the 10 countries with the highest rates of prematurity, with a rate of 11.2 per 100 live births^6^ .

Despite the high rate of prematurity in Brazil, there is a lack of data availability regarding recurrent prematurity and its possible associated factors, and therefore the rate of recurrent prematurity in the country is unknown. Thus, population-based studies to obtain these data are necessary because of the high financial costs that premature births generate for health systems, as well as their consequences for infant health, which include higher risks of neonatal and infant mortality^7^ , cardiac, renal, and cognitive changes during adulthood^8^ .

Different factors can affect the estimate of the recurrent prematurity rate, including gestational age limits, the occurrence of multiple gestations and spontaneous deliveries and by obstetric intervention^9^ . Studies show higher risks of recurrence of prematurity around the same gestational age and the same type of delivery as in the previous pregnancy, evidencing a dependency relationship between births^4 , 10^ .

Other factors associated with recurrent prematurity have been described in international studies, such as black race/color^11^ , delivery intervals shorter than two years^4^ , teenage pregnancy^12^ and advanced maternal age^13^ , low socioeconomic status^12^ , complications of the current pregnancy^12^ and lack of prenatal care^14^ . However, the associations differ according to the type of delivery.

Considering the high rates of prematurity in Brazil and the scarcity of national data regarding its recurrence, the objective of this study was to describe and estimate the rate of recurrent prematurity in Brazil according to the type of delivery, weighted by associated factors.

## METHODS

This study is part of the national “Birth in Brazil” survey, conducted between 2011 and 2012. “Birth in Brazil” was a hospital-based study that sought to evaluate prenatal care to delivery and postpartum care of women with hospital deliveries having as the pregnancy outcome a live newborn with any weight and gestational age (GA), or a dead fetus with weight greater than or equal to 500 grams and/or GA greater than 22 weeks.

The sample selection of the original study was composed of three stages. The first stage is the selection of hospitals by means of probability proportional to size (PPS). Thus, all hospitals with 500 or more deliveries/year in 2007, according to data from the information system on live births (Sinasc - *Sistema de Informação Sobre Nascidos Vivos* ), were selected and stratified by the five macroregions of the country. Finally, 266 hospitals were sampled, representing 19% of all those with 500 births or more in 2007. The second stage consisted of applying the inverse sampling method to ensure the minimum number of seven days of data collection necessary to reach the number of 90 postpartum women in each hospital. In the third and last stage, we selected eligible postpartum women to interviews. The final sample size was 23,894 postpartum women, with 90 interviews per hospital. Vasconcellos et al.^15^ present more details about the sample design and selection of postpartum women.

We extracted the data from face-to-face interviews with postpartum women during hospitalization; from prenatal care cards; and from maternal and newborn (NB) records. In addition, we conducted two telephone interviews after the puerperal women hospital discharge (six and twelve months after the hospital interview). Professionals trained by the central coordination team, using instruments developed specifically for this research, performed all data collection. A previous study by do Carmo Leal et al.^16^ gives more information about data collection.

This analysis included multiparous women with single gestation whose pregnancy outcome was a live preterm (< 37 weeks) or full term (39–40 weeks) newborn. We excluded early term neonates (37–38 weeks), since they have an increased risk for Neonatal Intensive Care Unit (NICU) admission and higher risks for neonatal morbidities^17^ . The estimation of GA was based primarily on the ultrasound performed between 7 and 13 weeks of gestation. In the absence of an ultrasound, the GA was based on the information reported by the puerperal woman in the interview and, finally, on the date of the last menstrual period and the birth weight percentile^18^ .

For the purposes of analysis, we categorized recurrent prematurity according to the type of delivery. We considered spontaneous delivery in cases of premature rupture of the fetal amniotic membranes (pPROM) or spontaneous onset of labor; and provider-initiated delivery when induction of labor was by means of drug intervention or by performing an elective cesarean section before the 37th week of gestation^19^ . Furthermore, early prematurity were considered to be all newborns with gestational age less than or equal to 33 weeks, and late prematurity were all those born between 34 and 36 weeks of gestation.

The primary exposure of interest was previous prematurity, extracted from the maternal record, prenatal care card, and interview with the woman. We used other covariates for the analysis, namely: type of hospital (public; mixed; private), maternal age (12–19 years; 20–34 years; ≥ 35 years), economic status according to the Brazilian Association of Market Research Institutes (classes A/B - high, C - middle, D/E - low), adequacy of prenatal care according to the modified Kotelchuck Index^20^ (inadequate/partially adequate; adequate/more than adequate), smoking in the third trimester of pregnancy (no; yes, less than 10 cigarettes per day; yes, 10 or more cigarettes per day), pregestational body mass index (BMI) (< 18.5; 18.5–24.9; 25.0–29.9; ≥ 30.0), parity (1–2 previous deliveries; ≥ 3 previous deliveries), previous cesarean section (no; yes), previous stillbirth or neonatal death (no; yes), malformation of current pregnancy (no; yes), chronic hypertension (no; yes), chronic diabetes (no; yes), hypertensive syndromes (hypertension, preeclampsia and HELLP syndrome), gestational diabetes (no; yes), other chronic disease (chronic heart disease other than hypertension, chronic kidney disease, and autoimmune disease), infection on admission for delivery (including urinary tract infection and other serious infections such as chorioamnionitis and pneumonia), premature placental abruption (no; yes), placenta previa (no; yes), and intrauterine growth restriction (IUGR) (no; yes).

We performed the data analysis in five steps. Initially, we constructed two directed acyclic graphs (DAG)^a^ , based on the literature, in order to identify the adjustment covariates required to estimate the association between previous prematurity and spontaneous recurrent prematurity, and by obstetric intervention.

The second step consisted of calculating the recurrent prematurity rate, where the total number of recurrent premature babies was divided by the total number of women with previous prematurity, multiplied by 100. Sequentially, we performed a descriptive analysis of the care, sociodemographic and obstetric characteristics of preterm and full-term infants, according to previous prematurity. We also performed a descriptive analysis of recurrent prematurity, categorized as spontaneous and by obstetric intervention, using full-term newborns as the reference group. At this stage, we used the chi-square test with Rao-Scott adjustment to compare proportions between groups.

For the third stage, we associated the adjustment covariates, initially flagged in the DAG, with recurrent spontaneous recurrent prematurity and by obstetric intervention by means of univariate logistic regression, using full-term newborns as the reference group. We expressed the results as odds ratios (OR), with their respective 95% confidence intervals (95%CI).

Then, we applied the propensity score method to estimate the causal effects of spontaneous recurrent prematurity and by obstetric intervention, taking full term newborns as the reference group. This strategy is usual in observational studies in order to reduce selection bias, because it enables a situation similar to that of quasi-experimental studies and therefore achieves a balance between treatment and control groups by adjustment variables^21^ , signaled by the DAG. For this, we calculated weights and used them to weight the groups using the average treatment effect (ATE). We also checked the balancing of the groups according to the adjustment covariates, using the absolute standardized difference of means. We considered balancing as adequate when this measure was less than 0.10^21^ .

Finally, we analyzed recurrent prematurity by the unconditional logistic regression model weighted by propensity score. We presented the results as crude odds ratios and adjusted odds ratios after balancing, with their 95%CI. We performed the analyses in R software version 3.4.3 (The R foundation for statistical computing).

During statistical analysis, we considered the complex sampling design using data weighting and calibration, and incorporating the design effect in order to ensure that the distribution of sampled puerperal women was similar to that observed in the population for the year 2011.

The research ethics committee of the *Escola Nacional de Saúde Pública Sergio Arouca* , *Fundação Oswaldo Cruz* (ENSP/Fiocruz), under the report no. 92/2010, approved the study “Birth in Brazil”. For the purpose of this study, the ethics committee approved the study under the report no. 2.972.153.

## RESULTS

We analyzed 6,701 newborns, of which 830 (12.4%) were from women with previous prematurity. The rate of recurrent prematurity was 42.0%, considering all women with previous prematurity. Among the 349 recurrent prematurity, 31.0% were early, 69.0% were late, 62.2% were spontaneous, and 37.8% were provider-initiated.

Recurrent prematurity, when compared to non-recurrent, were more frequent in women with A/B and C class socio-economic conditions, with three or more previous births, and with occurrence of stillbirth or neonatal death. Among full-term newborns, we found higher proportions of previous prematurity among women who were eutrophic and overweight, who had three or more previous deliveries, previous cesarean sections, an occurrence of stillbirth or neonatal death, with hypertensive syndromes, infection on admission for delivery and placenta previa, compared to full-term newborns without previous prematurity ( Table 1 ).

**Table 1:** Maternal and childbirth care characteristics used for weighting, according to previous prematurity. Brazil, 2011–2012.

	Premature (n = 1,215)		p^a^	Full term (n = 5,486)		p^a^
	
	Previous Prematurity			Previous Prematurity		
	
	Yes	No		Yes	No	
Total	349 (100.0)	866 (100.0)		481 (100.0)	5.005 (100.0)	
Type of hospital						
Public	190 (54.4)	420 (48.5)	0.172	224 (46.6)	2.157 (43.1)	0.257
Mixed	121 (34.7)	338 (39.0)		213 (44.3)	2.305 (46.1)	
Private	38 (10.9)	108 (12.5)		44 (9.1)	543 (10.8)	
Maternal age^b^						
12 to 19 years	31 (9.0)	70 (8.1)	0.310	27 (5.6)	313 (6.3)	0.931
20 to 34 years	257 (74.3)	618 (71.4)		383 (79.6)	3.972 (79.4)	
≥ 35 years	58 (16.8)	178 (20.6)		71 (14.8)	720 (14.4)	
Socioeconomic status^b^						
Class D/E – low	97 (27.9)	222 (25.8)	0.048	130 (27.2)	1.248 (25.2)	0.438
Class C – middle	165 (47.6)	471 (54.9)		251 (52.5)	2.597 (52.3)	
Class A/B – high	85 (24.5)	166 (19.3)		97 (20.3)	1.115 (22.5)	
Adequacy of prenatal care^b^						
Inadequate or partially adequate	142 (41.5)	356 (42.5)	0.762	178 (37.6)	1.864 (38.0)	0.925
Adequate or more than adequate	200 (58.5)	482 (57.5)		296 (62.4)	3.044 (62.0)	
Smoking in the third trimester of pregnancy^b^						
No	319 (91.7)	778 (89.8)	0.490	440 (91.5)	4.572 (91.3)	0.553
Yes, < 10 cigarettes/day	19 (5.5)	51 (5.9)		27 (5.6)	247 (4.9)	
Yes, ≥ 10 cigarettes/day	10 (2.9)	37 (4.3)		14 (2.9)	186 (3.7)	
BMI						
< 18.5	27 (7.7)	52 (6.0)	0.743	23 (4.8)	295 (5.9)	0.011
18.5–24.9	191 (54.7)	484 (55.9)		246 (51.1)	2.845 (56.8)	
25.0–29.9	91 (26.1)	231 (26.7)		150 (31.2)	1.296 (25.9)	
≥ 30.0	40 (11.5)	99 (11.4)		62 (12.9)	569 (11.4)	
Parity						
1–2 previous deliveries	238 (68.2)	691 (79.8)	0.001	363 (75.3)	4.149 (82.9)	< 0.001
≥ 3 previous deliveries	111 (31.8)	175 (20.2)		118 (24.7)	856 (17.1)	
Previous cesarean section^b^						
Yes	138 (39.5)	328 (38.1)	0.604	232 (48.6)	1.951 (39.2)	< 0.001
No	211 (60.5)	534 (61.9)		245 (51.4)	3.027 (60.8)	
Previous stillbirth or neonatal death^b^						
Yes	81 (23.2)	65 (7.5)	< 0.001	84 (17.5)	220 (4.4)	< 0.001
No	268 (76.8)	800 (92.5)		397 (82.5)	4.785 (95.6)	
Malformation ^b^						
Yes	13 (3.7)	33 (3.8)	0.944	8 (1.7)	59 (1.2)	0.359
No	336 (96.3)	833 (96.2)		473 (98.3)	4.946 (98.8)	
Chronic hypertension^b^						
Yes	27 (7.7)	49 (5.7)	0.176	21 (4.4)	155 (3.1)	0.141
No	322 (92.3)	817 (94.3)		460 (95.6)	4.850 (96.9)	
Chronic diabetes^b^						
Yes	6 (1.7)	26 (3.0)	0.206	6 (1.2)	64 (1.3)	0.953
No	343 (98.3)	840 (97.0)		475 (98.8)	4.941 (98.7)	

Clinical and obstetric complications						
Hypertensive syndromes^c^	84 (24.1)	173 (20.0)	0.114	85 (17.7)	472 (9.4)	< 0.001
Gestational diabetes	38 (10.9)	95 (11.0)	0.967	52 (10.8)	464 (9.3)	0.275
Other severe chronic disease^d^	6 (1.7)	7 (0.8)	0.163	5 (1.0)	41 (0.8)	0.613
Infection on admission for delivery	4 (1.1)	10 (1.2)	0.994	6 (1.2)	9 (0.2)	< 0.001
Premature placental abruption	22 (6.3)	40 (4.6)	0.227	8 (1.7)	53 (1.1)	0.229
Placenta previa	4 (1.1)	19 (2.2)	0.225	6 (1.2)	19 (0.4)	0.007
Restricted intrauterine growth	37 (10.6)	78 (9.0)	0.390	20 (4.2)	157 (3.1)	0.226

^a^ Rao-Scott χ^2^ test.

^b^ Different total due to missing values.

^c^ Hypertension, preeclampsia and HELLP syndrome.

^d^ Chronic heart disease (except hypertension), chronic kidney disease, and autoimmune diseases.

Table 2 shows that recurrent spontaneous preterm birth were more frequent in public hospitals and in adolescents, middle class, with low birth weight and eutrophic, with inadequate or partially adequate prenatal care, with three or more previous deliveries, without previous cesarean sections, with previous stillbirth or neonatal death, malformation, gestational diabetes, infection on admission for delivery, and premature placental abruption, when compared to full-term newborns. In contrast, recurrent provider-initiated preterm birth occurred more in women aged ≥ 35 years, high socioeconomic class, low birth weight or obese, adequate or more than adequate prenatal care, with previous cesarean section, previous stillbirth or neonatal death, and chronic hypertension, when compared to full-term newborns. Moreover, the recurrent provider-initiated preterm birth presented most of the clinical and obstetric complications, except for severe chronic disease and placenta previa.

**Table 2:** Type of recurrent prematurity according to maternal and birth care characteristics. Brazil, 2011–2012.

	Recurrent Prematurity				39–40 weeks (Ref.) n (%)

	Spontaneous^b^	p^a^	Provider-initiated	p^a^	
	
	n (%)		n (%)		
Total	**217 (100.0)**		**132 (100.0)**		**5,486 (100.0)**
Type of hospital					
Public	134 (61.8)	< 0.001	57 (43.2)	0.091	2,381 (43.4)
Mixed	68 (31.3)		53 (40.2)		2,518 (45.9)
Private	15 (6.9)		22 (16.7)		587 (10.7)
Maternal Age^b^					
12 to 19 years	28 (13.1)	< 0.001	3 (2.3)	0.007	340 (6.2)
20 to 34 years	159 (74.3)		98 (74.2)		4,355 (79.4)
≥ 35 years	27 (12.6)		31 (23.5)		791 (14.4)
Socioeconomic status^c^					
Class D/E – low	67 (30.9)	0.163	30 (22.7)	< 0.001	1,378 (25.3)
Class C – middle	113 (52.1)		54 (40.9)		2,848 (52.4)
Class A/B – high	37 (17.0)		48 (36.4)		1,212 (22.3)
Adequacy of prenatal care^c^					
Inadequate or partially adequate	106 (50.2)	0.001	37 (28.2)	0.011	2,042 (37.9)
Adequate or more than adequate	105 (49.8)		94 (71.8)		3,340 (62.1)
Smoking in the third trimester of pregnancy					
No	194 (89.4)	0.488	126 (95.4)	0.291	5,012 (91.3)
Yes, < 10 cigarettes/day	15 (6.9)		5 (3.8)		274 (5.0)
Yes, ≥ 10 cigarettes/day	8 (3.7)		1 (0.8)		200 (3.6)
BMI					
< 18.5	16 (7.4)	0.037	11 (8.3)	0.002	318 (5.8)
18.5–24.9	132 (60.8)		59 (44.7)		3,091 (56.3)
25.0–29.9	57 (26.3)		34 (25.8)		1,446 (26.4)
≥ 30.0	12 (5.5)		28 (21.2)		631 (11.5)
Parity					
1–2 previous deliveries	139 (64.1)	< 0.001	99 (75.0)	0.084	4,512 (82.2)
≥ 3 previous deliveries	78 (35.9)		33 (25.0)		974(17.8)
Previous cesarean section^c^					
Yes	57 (26.3)	< 0.001	81 (61.4)	< 0.001	2,183 (40.0)
No	160 (73.7)		51 (38.6)		3,272 (60.0)
Previous stillbirth or neonatal death					
Yes	48 (22.1)	< 0.001	33 (25.0)	< 0.001	304 (5.5)
No	169 (77.9)		99 (75.0)		5,182 (94.5)
Malformation					
Yes	8 (3.7)	0.020	5 (3.8)	0.058	67 (1.2)
No	209 (96.3)		127 (96.2)		5,419 (98.8)
Chronic hypertension					
Yes	3 (1.4)	0.061	24 (18.2)	< 0.001	176 (3.2)
No	214 (98.6)		108 (81.8)		5,310 (96.8)
Chronic diabetes					
Yes	4 (1.8)	0.694	2 (1.5)	0.995	70 (1.3)
No	213 (98.2)		130 (98.5)		5,416 (98.7)
Clinical and obstetric complications					
Hypertensive syndromes^d^	17 (7.8)	0.049	67 (50.8)	< 0.001	557 (10.1)
Gestational diabetes	11 (5.1)	0.019	27 (20.5)	< 0.001	516 (9.4)
Other severe chronic diseased ^e^	4 (1.8)	0.123	2 (1.5)	0.430	46 (0.8)
Infection on admission for delivery	3 (1.4)	0.030	2 (1.5)	0.056	15 (0.3)
Premature placental abruption	8 (3.7)	0.039	14 (10.6)	< 0.001	61 (1.1)
Placenta previa	2 (0.9)	0.714	2 (1.5)	0.272	25 (0.5)
Restricted intrauterine growth	7 (3.2)	0.414	29 (22.0)	< 0.001	177 (3.2)

Ref.: reference.

^a^ Rao-Scott χ^2^ test.

^b^ Spontaneous labor onset or premature rupture of membranes.

^c^ Different total due to missing values.

^d^ Hypertension, preeclampsia and HELLP syndrome.

^e^ Chronic heart disease (except hypertension), chronic kidney disease, and autoimmune diseases.

Multiple analysis showed higher odds of spontaneous recurrent prematurity in adolescents, those of lower class, and those who smoked 10 or more cigarettes per day in the third trimester of pregnancy. On the other hand, women with maternal age ≥ 35 years, of high socioeconomic class, with previous cesarean section, chronic hypertension and chronic diabetes had higher chances of recurrent provider-initiated preterm birth compared to full term newborns ( Table 3 ).

**Table 3:** Maternal characteristicsa used to weight women according to the type of recurrent prematurity. Brazil, 2011–2012.

	Spontaneous recurrent preterm birth^b^ (n = 197)	p^c^	Recurrent provider-initiated preterm birth^b^ (n = 136)	p^c^
	
	Crude OR (95%CI)		Crude OR (95%CI)	
Maternal age				
12 to 19 years	2.08 (1.60–2.71)	< 0.001	0.76 (0.47–1.24)	0.991
20 to 34 years	1.00		1.00	
≥ 35 years	1.02 (0.81–1.27)	0.912	1.74 (1.39–2.19)	< 0.001
Socioeconomic classification				
Class D/E – low	1.00		1.00	
Class C – middle	0.73 (0.61–0.88)	< 0.001	1.29 (1.00–1.68)	0.063
Class A/B – high	0.51 (0.40–0.64)	< 0.001	1.89 (1.43–2.51)	< 0.001
Smoking in the third trimester of pregnancy				
No	1.00		-	
Yes, < 10 cigarettes/day	1.36 (0.98–1.90)	0.070	-	-
Yes, ≥ 10 cigarettes/day	1.74 (1.22–2.47)	0.002	-	-
Parity				
1–2 previous deliveries	1.00		1.00	
≥ 3 previous deliveries	1.43 (1.18–1.73)	< 0.001	1.32 (1.04–1.67)	0.023
Previous cesarean section				
No	-	-	1.00	
Yes	-	-	2.30 (1.89–2.81)	< 0.001
Previous stillbirth or neonatal death				
No	1.00		1.00	
Yes	2.21 (1.70–2.87)	< 0.001	2.82 (2.11–3.76)	< 0.001
Chronic hypertension				
No	1.00		1.00	
Yes	1.01 (0.63–1.59)	0.990	3.06 (2.12–4.40)	< 0.001
Chronic diabetes				
No	1.00		1.00	
Yes	1.31 (0.67–2.57)	0.447	3.09 (1.74–5.49)	< 0.001

*OR: odds ratio;* 95%CI: 95% confidence interval.

^a^ All variables were selected based on the DAG (directed acyclic graph).

^b^ Different due to missing values.

^c^ The outcomes were compared with the category: 39–40 weeks gestation.

Table 4 shows the balancing that we performed before and after the propensity score, using standardized differences between the means of the groups. Before balancing, stillbirth or previous neonatal death (0.422) was the largest mean difference found for both groups. After weighting, we found the standardized differences between the means of the two groups approached zero for all covariates, indicating that the balancing after adjustment by the propensity score was adequate.

**Table 4:** Difference in means for the characteristics used in weighting women, according to the type of recurrent prematurity. Brazil, 2011–2012.

	Recurrent spontaneous preterm birth		Recurrent provider-initiated preterm birth	
	
	Before balancing	After balancing	Before balancing	After balancing
Maternal age				
12 to 19 years	0.034	-0.026	0.034	-0.026
20 to 34 years	-0.021	0.009	-0.021	0.009
≥ 35 years	-0.000	0.026	-0.000	0.026
Socioeconomic status				
Class D/E - low	0.044	0.050	0.044	0.050
Class C - middle	-0.018	-0.049	-0.018	-0.049
Class A/B - high	-0.025	0.004	-0.025	0.004
Smoking in the third trimester of pregnancy				
No	-0.015	0.035	-	-
Yes, < 10 cigarettes/day	0.042	-0.027	-	-
Yes, ≥ 10 cigarettes/day	-0.031	-0.021	-	-
Parity				
1–2 previous deliveries	-0.222	-0.056	-0.222	-0.056
≥ 3 previous deliveries	0.222	0.056	0.222	0.056
Previous cesarean section				
Yes	-	-	0.089	-0.018
No	-	-	-0.089	0.018
Previous stillbirth or neonatal death				
Yes	0.422	-0.035	0.422	-0.035
No	-0.422	0.035	-0.422	0.035
Chronic hypertension				
Yes	0.099	0.002	0.099	0.002
No	-0.099	-0.002	-0.099	-0.002
Chronic Diabetes				
Yes	0.005	-0.003	0.005	-0.003
No	-0.005	0.003	-0.005	0.003

Given this, the final analysis showed that women with previous prematurity have 3.89 times the chance of having spontaneous recurrent prematurity (ORaj: 3.89; 95%CI 3.01–5.03) and 3.47 times the chance of having recurrent provider-initiated preterm birth (ORaj: 3.47; 95%CI 2.59–4.66) when compared to women with full-term newborns ( Table 5 ).

**Table 5:** Crude and adjusted odds ratios when comparing recurrent preterm with full-term newborns, after propensity score. Brazil, 2011–2012.

	Crude OR (95%CI)	OR after balancing (95%CI)
Recurrent prematurity		
General		
< 37 weeks	4.25 (3.62–4.97)	3.72 (3.01–4.61)
39–40 weeks	1.00	
Spontaneous		
< 37 weeks	4.10 (3.39–4.95)	3.89 (3.01–5.03)
39–40 weeks	1.00	1.00
Provider-initiated		
< 37 weeks	4.48 (3.59–5.60)	3.47 (2.59–4.66)
39–40 weeks	1.00	1.00

OR: odds ratio; 95%CI: 95% confidence interval.

## DISCUSSION

The rate of recurrent prematurity was 42.0% among women with previous prematurity, most of which was late and of spontaneous cause. Factors related to social vulnerability showed higher odds for spontaneous recurrent prematurity, while better socioeconomic conditions were associated with recurrent provider-initiated preterm birth. In addition, previous prematurity increased the chances of recurrence of spontaneous and provider-initiated preterm births.

The recurrence rate in our study was higher than those reported in studies conducted in the Netherlands (29.3%)^22^ , Japan (22.3%)^23^ and Utah (21.0%)^12^ . The reasons for this are still poorly understood, however, studies show that socioeconomic factors, inadequate prenatal care, maternal risk behaviors, obstetric complications, genetic factors and models of obstetric care are possible determinants of recurrent prematurity^4 , 12 , 24^ .

When analyzing recurrent prematurity by type of delivery, we found higher frequencies of spontaneous premature birth (62.2%). Moreover, adolescents with worse socioeconomic conditions were more likely to have spontaneous recurrent prematurity, while women with better socioeconomic conditions, prior cesarean section, chronic hypertension and chronic diabetes were significantly associated with recurrent provider-initiated preterm birth. These findings corroborate previous Brazilian studies identifying that women in situations of social vulnerability have higher risks of spontaneous prematurity, while those with better socioeconomic conditions have higher risks of prematurity by obstetric intervention^25 , 26^ . In addition, we observed significantly higher values of prematurity in underweight or obese women. Inadequate nutrition is closely related to the low socioeconomic status of pregnant women, just as overweight is associated with maternal complications (gestational diabetes and hypertensive syndromes). Therefore, gestational weight gain different from the recommended one leads to higher risks of adverse outcomes for mothers and their newborns^27 , 28^ .

This study also revealed higher chances of recurrence of spontaneous and provider-initiated preterm birth regardless of the type of previous prematurity. Retrospective cohort conducted in 20 hospitals located in Utah showed that previous spontaneous preterm is a strong predictor of subsequent spontaneous preterm birth (RRaj: 5.64; 95%CI 5.27–6.05), just as previous provider-initiated preterm has higher risks of recurrent provider-initiated preterm birth (RRaj: 9.10; 95%CI 4.68–17.71) and vice versa^29^ .

In Brazil, it is possible that women with previous prematurity by obstetric intervention have even higher risks of recurrence, due to the effects of the organization of obstetric care and women’s choice for the same type of delivery, especially cesarean section. Domingues et al.^30^ showed that multiparous women with previous cesarean sections have an initial preference for cesarean sections in subsequent pregnancies. Among the reasons for this choice, the study points out the possibility of scheduling a cesarean section at the very beginning of pregnancy^30^ . As a result, a study by Nakamura-Pereira et al.^31^ , using the Robson Classification, evidenced that multiparous women with prior cesarean section and cephalic presentation ≥ 37 weeks represent the second group that most contributes to cesarean section rates in Brazil. Another study by Nakamura-Pereira et al.^32^ also identified that among women eligible to attempt labor after a cesarean section, 66.1% had elective repeat cesarean sections, which demonstrates adherence to the saying “once a cesarean section, always a cesarean section”. These phenomena are intrinsically related to the increase in increasingly earlier deliveries, which contribute to nearly 10% of cesarean rates in Brazil^31^ .

In addition to elective cesarean section, maternal clinical complications also relate to provider-initiated preterm birth. Retrospective cohort conducted in Northern Tanzania showed that women who had preeclampsia in previous pregnancies had a 50% higher risk of recurrent prematurity compared to women with normal blood pressure^33^ . Therefore, the recommendation is to identify early women with a history of prematurity associated with comorbidities and treat them timely in the prenatal period and during labor to prevent negative maternal-fetal outcomes.

The number of previous prematurity, birth order, and gestational age^2 , 29 , 34^ influence the recurrence of prematurity. In a cohort of women with three consecutive singleton pregnancies, Hiersch et al.^2^ found RR = 3.1 (95% CI 1.9–4.9) for recurrent prematurity at third pregnancy in women who had prematurity only at first pregnancy; RR = 5.6 (95% CI 3.6–8.8) in women who had this outcome at second pregnancy; and RR = 38.2 (95% CI 20.6–70.8) in women with prematurity at the first two deliveries, when compared to women who had a full-term newborn. Therefore, recurrence in a third pregnancy is more associated with women with a history of prematurity in their second pregnancy than in their first^34^ . For gestational age, a retrospective cohort conducted in California found that women with a first birth before 32 weeks gestation had 23. 3 times higher risk of recurrence before 32 weeks gestation^35^ , so the earlier the previous birth, the higher the risk of recurrent prematurity.

Regarding interventions to prevent increasingly early births, Mazaki-Tovi et al.^9^ , in a literature review, state that the best strategy is still progesterone administration. Uterine cerclage is also possible, but only in the presence of uterine cervical insufficiency, or in women with a previous incidence of cervical insufficiency, or in women with early uterine cervical shortening diagnosed by ultrasound^9 , 36^ .

The highlight of this study was to estimate the chance of recurrent prematurity in multiparous women in Brazil based on the national survey “Birth in Brazil”, which used a representative sample of women considering the country’s regions, geographic location (capital or interior) and type of hospital care (private, public or mixed). Also highlighted was the method of analysis applied – propensity score weighting – allowing the results of this study to be brought closer to those of an experimental study, making the groups comparable and the results more robust.

However, this study has some limitations. Only puerperal women attending hospitals with more than 500 births/year (representing 80% of births in the country) were included and, therefore, it is possible that women with deliveries in smaller hospitals, or with home or public deliveries, have different risks for recurrent prematurity. It was also not possible to estimate the direct effect of the type of previous prematurity on the type of recurrent prematurity, due to the absence of information on previous pregnancies. In addition, it was not possible to analyze prematurity according to gestational age because of the low frequencies of newborns in each subgroup of recurrent prematurity. Future studies should include these factors for a complete investigation of the risks for recurrent spontaneous prematurity and by obstetric intervention.

In conclusion, previous prematurity proved to be a strong predictor for recurrence of spontaneous and provider-initiated preterm births. Unfortunately, Brazil is among the ten countries that together contribute to 60% of premature births in the world^37^ . Besides bringing implications for the child’s health, prematurity also represents the leading cause of neonatal death, and therefore Brazil faces the great challenge of reducing its prematurity rates. Thus, the findings of this study have important clinical implications for the monitoring and management of pregnant women with a history of prematurity, aiming to assist health care professionals to plan with adequate care for the prevention of new prematurity and to reduce the risk of adverse neonatal outcomes in this population.

## Footnotes

a Dias BAS. Recorrência da cesariana e da prematuridade na pesquisa Nascer no Brasil [tese]. Rio de Janeiro: Escola Nacional de Saúde Pública Sergio Arouca, Fundação Oswaldo Cruz; 2021 [cited 2022 Jan 6]. Available from: https://www.arca.fiocruz.br/bitstream/icict/49461/2/barbara_almeida_soares_dias_ensp_dout_2021.pdf

## References

[1] Phillips C, Velji Z, Hanly C, Metcalfe A. Risk of recurrent spontaneous preterm birth: a systematic review and meta-analysis. BMJ Open. 2017;7(6):e015402. 10.1136/bmjopen-2016-015402 PMC5734267 28679674

[2] Hiersch L, Pasternak Y, Melamed N, Meshulam M, Shashar R, Hadar E, et al. The risk of preterm birth in women with three consecutive deliveries: the effect of number and type of prior preterm births. J Clin Med. 2020;9(12):3933. 10.3390/jcm9123933 PMC7761894 33291626

[3] Tuon RA, Ambrosano GMB, Silva SMCV, Pereira AC. Impacto do monitoramento telefônico de gestantes na prevalência da prematuridade e análise dos fatores de risco associados em Piracicaba, São Paulo, Brasil. Cad Saude Publica. 2016;32(7):e00107014. 10.1590/0102-311X00107014 27462851

[4] Yamashita M, Hayashi S, Endo M, Okuno K, Fukui O, Mimura K, et al. Incidence and risk factors for recurrent spontaneous preterm birth: a retrospective cohort study in Japan. J Obstet Gynaecol Res 2015;41(11):1708-14. 10.1111/jog.12786 26311118

[5] March of Dimes; PMNCH; Save the Children; World Health Organization. Born too soon: the global action report on preterm birth. Geneva (CH): WHO; 2012.

[6] Chawanpaiboon S, Vogel JP, Moller AB, Lumbiganon P, Petzold M, Hogan D, et al. Global, regional, and national estimates of levels of preterm birth in 2014: a systematic review and modelling analysis. Lancet Glob Health. 2019;7(1):e37-46. 10.1016/S2214-109X(18)30451-0 PMC6293055 30389451

[7] França EB, Lansky S, Rego MAS, Malta DC, França JS, Teixeira R, et al. Principais causas da mortalidade na infância no Brasil, em 1990 e 2015: estimativas do estudo de Carga Global de Doença. Rev Bras Epidemiol. 2017;20 Supl 1:46-60. 10.1590/1980-5497201700050005 28658372

[8] Chehade H, Simeoni U, Guignard JP, Boubred F. Preterm birth: long term cardiovascular and renal consequences. Curr Pediatr Rev. 2018;14(4):219-26. 10.2174/1573396314666180813121652 PMC6416185 30101715

[9] Mazaki-Tovi S, Romero R, Kusanovic JP, Erez O, Pineles BL, Gotsch F, et al. Recurrent preterm birth. Semin Perinatol. 2007;31(3):142-58. 10.1053/j.semperi.2007.04.001 PMC3500643 17531896

[10] Ananth CV, Getahun D, Peltier MR, Salihu HM, Vintzileos AM. Recurrence of spontaneous versus medically indicated preterm birth. Am J Obstet Gynecol. 2006;195(3):643-50. 10.1016/j.ajog.2006.05.022 16949395

[11] Baer RJ, Yang J, Berghella V, Chambers CD, Coker TR, Kuppermann M, et al. Risk of preterm birth by maternal age at first and second pregnancy and race/ethnicity. J Perinat Med. 2018;46(5):539-46. 10.1515/jpm-2017-0014 28455952

[12] Simonsen SE, Lyon JL, Stanford JB, Porucznik CA, Esplin MS, Varner MW. Risk factors for recurrent preterm birth in multiparous Utah women: a historical cohort study. BJOG. 2013;120(7):863-72. 10.1111/1471-0528.12182 23418923

[13] Ouattara A, Ouedraogo CM, Ouedraogo A, Lankoande J. [Factors associated with preterm birth in an urban African environment: a case-control study at the University Teaching Hospital of Ouagadougou and Saint Camille Medical Center]. Med Sante Trop. 2015;25(3):296-9. French. 10.1684/mst.2015.0465 26102547

[14] Ratzon R, Sheiner E, Shoham-Vardi I. The role of prenatal care in recurrent preterm birth. Eur J Obstet Gynecol Reprod Biol. 2011;154(1):40-4. 10.1016/j.ejogrb.2010.08.011 20869804

[15] Vasconcellos MTL, Silva PLN, Pereira APE, Schilithz AOC, Souza Junior PRB, Szwarcwald CL. Desenho da amostra Nascer no Brasil: Pesquisa Nacional sobre Parto e Nascimento. Cad Saude Publica. 2014;30 Supl 1:S49-58. 10.1590/0102-311X00176013

[16] Leal MC, Silva AAM, Dias MAB, Gama SGN, Rattner D, Moreira ME, et al. Birth in Brazil: national survey into labour and birth. Reprod Health. 2012;9:15. 10.1186/1742-4755-9-15 PMC3500713 22913663

[17] Leal MC, Esteves-Pereira AP, Nakamura-Pereira M, Domingues RMSM, Dias MAB, Moreira ME, et al. Burden of early-term birth on adverse infant outcomes: a population-based cohort study in Brazil. BMJ Open. 2017;7(12):e017789. 10.1136/bmjopen-2017-017789 PMC5770827 29284716

[18] Pereira APE, Leal MC, Gama SGN, Domingues RMSM, Schilithz AOC, Bastos MH. Determinação da idade gestacional com base em informações do estudo Nascer no Brasil. Cad Saude Publica. 2014;30 Supl 1:S59-70. 10.1590/0102-311X00160313

[19] Goldenberg RL, Culhane JF, Iams JD, Romero R. Epidemiology and causes of preterm birth. Lancet. 2008;371(9606):75-84. 10.1016/S0140-6736(08)60074-4 PMC7134569 18177778

[20] Leal MC, Gama SGN, Ratto KMN, Cunha CB. Uso do índice de Kotelchuck modificado na avaliação da assistência pré-natal e sua relação com as características maternas e o peso do recém-nascido no Município do Rio de Janeiro. Cad Saude Publica. 2004;20 Supl 1:S63-72. 10.1590/S0102-311X2004000700007 16636736

[21] Silva AAM. Introdução à inferência causal em Epidemiologia: uma abordagem gráfica e contrafactual. Editora Fiocruz; 2021.

[22] Koullali B, Kamphuis E, Hof M, Robertson S, Pajkrt E, Groot C, et al. The effect of interpregnancy interval on the recurrence rate of spontaneous preterm birth: a retrospective cohort study. Am J Perinatol. 2016;34(2):174-82. 10.1055/s-0036-1584896 27367283

[23] Seyama R, Makino S, Nojiri S, Takeda J, Suzuki T, Maruyama Y, et al. Retrospective study of the recurrence risk of preterm birth in Japan. J Matern Fetal Neonatal Med. 2020;1–5. 10.1080/14767058.2020.1727435 32068466

[24] Grantz KL, Hinkle SN, Mendola P, Sjaarda LA, Leishear K, Albert PS. Differences in risk factors for recurrent versus incident preterm delivery. Am J Epidemiol. 2015;182(2):157-67. 10.1093/aje/kwv032 PMC4493980 26033931

[25] Leal MC, Esteves-Pereira AP, Nakamura-Pereira M, Torres JA, Theme-Filha M, Domingues RMSM, et al. Prevalence and risk factors related to preterm birth in Brazil. Reprod Health. 2016;13 Suppl 3:127. 10.1186/s12978-016-0230-0 PMC5073982 27766978

[26] Souza RT, Cecatti JG, Passini Jr R, Tedesco RP, Lajos GJ, Nomura ML, et al. The burden of provider-initiated preterm birth and associated factors: evidence from the Brazilian Multicenter Study on Preterm Birth (EMIP). PLoS One. 2016;11(2):e0148244. 10.1371/journal.pone.0148244 PMC4743970 26849228

[27] Silva JC, Amaral AR, Ferreira BS, Petry JF, Silva MR, Krelling PC. Obesidade durante a gravidez: resultados adversos da gestação e do parto. Rev Bras Ginecol Obstet. 2014;36(11):509-13. 10.1590/S0100-720320140005024 25493403

[28] Goldstein RF, Abell SK, Ranasinha S, Misso M, Boyle JA, Black MH, et al. Association of gestational weight gain with maternal and infant outcomes: a systematic review and meta-analysis. JAMA. 2017;317(21):2207-25. 10.1001/jama.2017.3635 PMC5815056 28586887

[29] Laughon SK, Albert PS, Leishear K, Mendola P. The NICHD Consecutive Pregnancies Study: recurrent preterm delivery by subtype. Am J Obstet Gynecol. 2014;210(2):131.e1-8. 10.1016/j.ajog.2013.09.014 PMC3934564 24036403

[30] Domingues RMSM, Dias MAB, Nakamura-Pereira M, Torres JA, d’Orsi E, Pereira APE, et al. Processo de decisão pelo tipo de parto no Brasil: da preferência inicial das mulheres à via de parto final. Cad Saude Publica. 2014;30 Supl 1:S101-16. 10.1590/0102-311X00105113

[31] Nakamura-Pereira M, Leal MC, Esteves-Pereira AP, Domingues RMSM, Torres JA, Dias MAB, et al. Use of Robson classification to assess cesarean section rate in Brazil: the role of source of payment for childbirth. Reprod Health. 2016;13 Suppl 3:128. 10.1186/s12978-016-0228-7 PMC5073850 27766941

[32] Nakamura-Pereira M, Esteves-Pereira AP, Gama SGN, Leal M. Elective repeat cesarean delivery in women eligible for trial of labor in Brazil. Int J Gynecol Obstet. 2018;143(3):351-9. 10.1002/ijgo.12660 30182481

[33] Kalengo NH, Sanga LA, Philemon RN, Obure J, Mahande MJ. Recurrence rate of preterm birth and associated factors among women who delivered at Kilimanjaro Christian Medical Centre in Northern Tanzania: a registry based cohort study. PLoS One. 2020;15(9):e0239037. 10.1371/journal.pone.0239037 PMC7489548 32925974

[34] Ouh YT, Park JH, Ahn KH, Hong SC, Oh MJ, Kim HJ, et al. Recurrent risk of preterm birth in the third pregnancy in Korea. J Korean Med Sci. 2018;33(24):e170. 10.3346/jkms.2018.33.e170 PMC5990443 29892210

[35] Yang J, Baer RJ, Berghella V, Chambers C, Chung P, Coker T, et al. Recurrence of preterm birth and early term birth. Obstet Gynecol. 2016;128(2):364-72. 10.1097/AOG.0000000000001506 PMC5055875 27400000

[36] Flood K, Malone FD. Prevention of preterm birth. Semin Fetal Neonat Med. 2012;17(1):58-63. 10.1016/j.siny.2011.08.001 21893439

[37] Chawanpaiboon S, Vogel JP, Moller AB, Lumbiganon P, Petzold M, Hogan D, et al. Global, regional, and national estimates of levels of preterm birth in 2014: a systematic review and modelling analysis. Lancet Glob Health. 2019;7(1):e37-46. 10.1016/S2214-109X(18)30451-0 PMC6293055 30389451

