# Supplementary material for: Recurrent preterm birth: data from the study “Birth in Brazil”
Source: Rev Saude Publica. 2022 Mar 4;56:7. doi: 10.11606/s1518-8787.2022056003527 (PMC8910113; doi:10.11606/s1518-8787.2022056003527)
Supplement: Translation

# Translation: Prematuridade recorrente: dados do estudo “Nascer no Brasil”

**Authors:** Barbara Almeida Soares Dias, Maria do Carmo Leal, Katrini Guidolini Martinelli, Marcos Nakamura-Pereira, Ana Paula Esteves-Pereira, Edson Theodoro dos Santos

**Affiliations:** I Brasil Fundação Oswaldo Cruz. Escola Nacional de Saúde Pública. Pós-Graduação em Epidemiologia em Saúde Pública. Rio de Janeiro, RJ, Brasil; II Brasil Fundação Oswaldo Cruz. Escola Nacional de Saúde Pública. Departamento de Epidemiologia e Métodos Quantitativos em Saúde. Rio de Janeiro, RJ, Brasil; III Brasil Universidade Federal do Espírito Santo. Programa de Pós-Graduação em Saúde Coletiva. Vitória, ES, Brasil; IV Brasil Fundação Oswaldo Cruz. Instituto Fernandes Figueira. Rio de Janeiro, RJ, Brasil; V Brasil Universidade Federal do Espírito Santo. Departamento de Medicina Social. Vitória, ES, Brasil

**Keywords:** Nascimento Prematuro, epidemiologia, Fatores de Risco, Pontuação de Propensão, História Reprodutiva, Inquéritos Epidemiológicos

## Abstract

**OBJETIVO:**

Descrever e estimar a taxa de prematuridade recorrente no Brasil segundo o tipo de parto, ponderado pelos fatores associados.

**MÉTODOS:**

Os dados foram obtidos do estudo nacional de base hospitalar “Nascer no Brasil”, realizado em 2011 e 2012, a partir de entrevistas com 23.894 mulheres. Inicialmente foi utilizado o teste qui-quadrado para verificar as diferenças entre os recém-nascidos, segundo a prematuridade prévia e o tipo de prematuridade recorrente. Sequencialmente, aplicou-se o método de ponderação pelo escore de propensão para equilibrar os grupos de acordo com as seguintes covariáveis: idade materna, classificação socioeconômica, tabagismo durante a gravidez, paridade, cesárea anterior, natimorto ou óbito neonatal anterior, hipertensão crônica e diabetes crônica. Por último, foi realizada regressão logística múltipla para estimar a prematuridade recorrente.

**RESULTADOS:**

Foram analisados 6.701 recém-nascidos. A taxa de prematuridade recorrente foi de 42,0%, considerando todas as mulheres com prematuridade prévia. Dentre os prematuros recorrentes, 62,2% foram espontâneos e 37,8% ocorreram por intervenção-obstétrica. Após a ponderação pelo escore de propensão, verificou-se que mulheres com prematuridade prévia têm 3,89 vezes a chance de terem prematuridade recorrente espontânea (ORaj = 3,89; IC95% 3,01–5,03) e 3,47 vezes a chance de terem prematuridade recorrente por intervenção obstétrica (ORaj = 3,47; IC95% 2,59–4,66), em comparação às mulheres que tiveram recém-nascidos termo completo.

**CONCLUSÕES:**

A prematuridade prévia revelou-se um forte preditor para sua recorrência. Assim, ampliar e melhorar o monitoramento e manejo de gestantes com história de prematuridade impacta fortemente na redução das taxas e, consequentemente, na redução dos riscos de morbimortalidade infantil no país.

## INTRODUÇÃO

A prematuridade recorrente ocorre quando se realiza dois ou mais partos antes de completar 37 semanas de gestação^1^ . Embora a sua etiologia seja complexa, multifatorial e até mesmo desconhecida, a literatura científica mostra que o histórico de prematuridade compreende um dos principais fatores para sua ocorrência em gestações subsequentes^1^ .

A taxa de prematuridade tem aumentado em todo o mundo, principalmente devido ao aumento de nascimentos prematuros tardios, frequentemente associados às intervenções obstétricas^5^ . Em 2014, a taxa global de prematuridade foi de 10,6 por 100 nascidos vivos, sendo a Ásia responsável por 52,9% desses nascimentos. O Brasil ocupa o nono lugar no ranking dos 10 países com as maiores taxas de prematuridade, com uma taxa de 11,2 por 100 nascidos vivos^6^ .

Apesar da elevada taxa de prematuridade no Brasil, verifica-se uma falta de disponibilidade de dados relativos à prematuridade recorrente e os seus possíveis fatores associados e, portanto, a taxa de prematuridade recorrente no país é desconhecida. Dessa forma, estudos de base populacional para a obtenção desses dados são necessários, em virtude dos altos custos financeiros que partos prematuros geram para os sistemas de saúde, assim como as suas consequências para a saúde infantil, que incluem maiores riscos de mortalidade neonatal e infantil^7^ e alterações cardíacas, renais e cognitivas durante a vida adulta^8^ .

Diferentes fatores podem afetar a estimativa da taxa de prematuridade recorrente, que englobam os limites de idade gestacional, a ocorrência de gestações múltiplas e de partos espontâneos e por intervenção obstétrica^9^ . Assim, estudos mostram maiores riscos de recorrência de prematuridade em torno da mesma idade gestacional e do mesmo tipo de parto da gestação anterior, evidenciando uma relação de dependência entre os nascimentos^4 , 10^ .

Outros fatores associados à prematuridade recorrente foram descritos em estudos internacionais, tais como raça/cor preta^11^ , intervalo interpartal menor que dois anos^4^ , gravidez na adolescência^12^ e em idade materna avançada^13^ , baixa condição socioeconômica^12^ , complicações da gestação atual^12^ e falta de assistência pré-natal^14^ ; todavia, as associações diferem segundo o tipo de parto.

Considerando as altas taxas de prematuridade no Brasil e a escassez de dados nacionais referente a sua recorrência, o objetivo deste estudo foi descrever e estimar a taxa de prematuridade recorrente no Brasil segundo o tipo de parto, ponderado pelos fatores associados.

## MÉTODOS

Este estudo é parte da pesquisa nacional “Nascer no Brasil”, realizada entre 2011 e 2012. “Nascer no Brasil” foi um estudo de base hospitalar que buscou avaliar a assistência pré-natal ao parto e ao pós-parto de mulheres com parto hospitalar que tiveram como desfecho da gestação um recém-nascido vivo com qualquer peso e idade gestacional (IG), ou um feto morto com peso maior ou igual a 500 gramas e/ou IG maior que 22 semanas.

A seleção da amostra do estudo original foi composta por três etapas. A primeira etapa constitui-se pela seleção dos hospitais por meio de probabilidade proporcional ao tamanho (PPT). Desse modo, todos os hospitais com 500 ou mais partos/ano em 2007 segundo dados do Sistema de Informação sobre Nascidos Vivos (Sinasc) foram selecionados e estratificados pelas cinco macrorregiões do país. Ao final, um total de 266 hospitais foi amostrado, representando 19% de todos aqueles com 500 nascimentos ou mais em 2007. A segunda etapa consistiu na aplicação do método de amostragem inversa para assegurar o número mínimo de sete dias de coleta de dados, necessário para atingir o número de 90 puérperas em cada hospital. Na terceira e última etapa, foram selecionadas as puérperas elegíveis a serem entrevistadas. O tamanho final da amostra foi de 23.894 puérperas, com 90 entrevistas por hospital. Demais detalhes acerca do desenho amostral e seleção das puérperas podem ser encontrados em Vasconcellos et al.^15^ .

Os dados foram extraídos das entrevistas face a face com as puérperas durante a internação hospitalar; dos cartões de pré-natal; e dos prontuários maternos e dos recém-nascidos (RN). Além disso, foram aplicadas duas entrevistas telefônicas após a alta hospitalar das puérperas (seis e doze meses após a entrevista hospitalar). Toda a coleta de dados foi realizada por profissionais treinados pela equipe de coordenação central utilizando instrumentos desenvolvidos especificamente para esta pesquisa. Maiores informações acerca da coleta de dados podem ser obtidas em estudo prévio de do Carmo Leal et al.^16^ .

Nesta análise foram incluídas multíparas com gestação única cujo desfecho da gestação foi um recém-nascido vivo prematuro (< 37 semanas) ou termo completo (39–40 semanas). Recém-nascidos termo precoce (37–38 semanas) foram excluídos, uma vez que têm um risco aumentado para admissão em Unidade de Terapia Intensiva Neonatal (UTIN) e maiores riscos para morbidades neonatais^17^ . A estimativa da IG baseou-se prioritariamente na ultrassonografia realizada entre sete e 13 semanas de gestação. Na ausência de ultrassonografia, a IG baseou-se nas informações relatadas pela puérpera na entrevista e, por último, na data da última menstruação e no percentil de peso ao nascer^18^ .

Para análise da prematuridade recorrente foi realizada a sua categorização de acordo com o tipo de parto. Dessa forma, considerou-se parto espontâneo quando houve ruptura prematura das membranas fetais amnióticas (pPROM) ou início espontâneo do trabalho de parto; e parto por intervenção obstétrica quando a indução do trabalho de parto foi por meio de intervenção medicamentosa ou realização de uma cesariana eletiva antes da 37ª semana de gestação^19^ . Ademais, considerou-se prematuros precoces todos os recém-nascidos com idade gestacional menor ou igual a 33 semanas e prematuros tardios todos aqueles nascidos entre 34 e 36 semanas de gestação.

A exposição primária de interesse foi a prematuridade prévia, extraída do prontuário materno, cartão de pré-natal e entrevista com a puérpera. Outras covariáveis foram utilizadas para a análise, a saber: tipo de hospital (público; misto; privado), idade materna (12–19 anos; 20–34 anos; ≥ 35 anos), classificação econômica segundo a Associação Brasileira de Institutos de Pesquisa de Mercado (classes A/B – alta, C – média, D/E – baixa), adequação do pré-natal segundo o Índice de Kotelchuck modificado^20^ (inadequado/parcialmente adequado; adequado/mais que adequado), fumo no terceiro trimestre da gestação (não; sim, menos de 10 cigarros por dia; sim, 10 ou mais cigarros por dia), índice de massa corporal (IMC) pré-gestacional (< 18,5; 18,5–24,9; 25,0–29,9; ≥ 30,0), paridade (1–2 partos anteriores; ≥ 3 partos anteriores), cesariana prévia (não; sim), natimorto ou óbito neonatal anterior (não; sim), malformação da gestação atual (não; sim), hipertensão crônica (não; sim), diabetes crônica (não; sim), síndromes hipertensivas (hipertensão, pre-eclâmpsia e síndrome HELLP), diabetes gestacional (não; sim), outra doença crônica (doenças cardíacas crônicas que não a hipertensão, doenças renais crônicas e doenças auto-imunes), infecção na admissão para o parto (incluindo infecção do trato urinário e outras infecções graves, como corioamnionite e pneumonia), descolamento prematuro da placenta (não; sim), placenta prévia (não; sim) e crescimento intrauterino restrito (CIUR) (não; sim).

A análise dos dados foi feita em cinco etapas. Inicialmente foram construídos dois gráficos acíclicos direcionados (DAG)^a^ , com base na literatura, a fim de identificar as covariáveis de ajuste necessárias para estimar a associação entre prematuridade prévia e prematuridade recorrente espontânea e por intervenção obstétrica.

A segunda etapa consistiu no cálculo da taxa de prematuridade recorrente, em que se dividiu o total de prematuros recorrentes pelo total de mulheres com prematuridade prévia, multiplicado por 100. Sequencialmente, realizou-se a análise descritiva das características assistenciais, sociodemográficas e obstétricas dos prematuros e termos completos, segundo a prematuridade prévia. Também foi realizada a análise descritiva dos prematuros recorrentes, categorizados em espontâneos e por intervenção obstétrica, utilizando como grupo de referência os recém-nascidos termo completo. Nessa etapa foi utilizado o teste Qui-quadrado com ajuste de Rao-Scott para comparar as proporções entre os grupos.

Para a terceira etapa, as covariáveis de ajuste, sinalizadas inicialmente nos DAG, foram associadas à prematuridade recorrente espontânea e por intervenção obstétrica por meio de regressão logística univariada, utilizando como grupo de referência os recém-nascidos termo completo. Os resultados foram expressos como razões de chance (OR), com os respectivos intervalos de 95% de confiança (IC95%).

Em seguida, aplicou-se o método de ponderação pelo escore de propensão para estimar os efeitos causais da prematuridade recorrente espontânea e por intervenção obstétrica, tomando como grupo de referência os recém-nascidos termo completo. Essa estratégia é utilizada em estudos observacionais com o objetivo de reduzir viés de seleção, pois possibilita uma situação semelhante à dos estudos quase-experimentais e, portanto, obtém um equilíbrio entre os grupos de tratamento e controle por variáveis de ajuste^21^ , sinalizadas pelo DAG. Para isso, foram calculados pesos, que foram utilizados na ponderação dos grupos por meio do efeito médio do tratamento (ATE). Também se verificou o balanceamento dos grupos segundo as covariáveis de ajuste, utilizando a diferença padronizada absoluta das médias. O balanceamento foi considerado adequado quando essa medida foi menor que 0,10^21^ .

Por último, a prematuridade recorrente foi analisada pelo modelo de regressão logística não condicional, ponderado pelo escore de propensão. Os resultados foram apresentados como razões de chances brutas e razões de chances ajustadas após o balanceamento, com os respectivos IC95%. As análises foram realizadas no *software R* versão 3.4.3 ( *The R Foundation for Statistical Computing* ).

Vale destacar que durante toda análise estatística foi considerado o desenho complexo da amostragem com utilização de ponderação e calibração dos dados e incorporação do efeito de desenho, a fim de assegurar que a distribuição das puérperas amostradas fosse semelhante à observada na população para o ano de 2011.

O estudo “Nascer no Brasil” foi aprovado pelo Comitê de Ética em pesquisa da Escola Nacional de Saúde Pública Sergio Arouca, Fundação Oswaldo Cruz (ENSP/Fiocruz), sob o parecer nº 92/2010. Para o objetivo deste estudo, houve aprovação do referido Comitê de Ética sob o parecer nº 2.972.153.

## RESULTADOS

Foram analisados 6.701 recém-nascidos, dos quais 830 (12,4%) foram de mulheres com prematuridade prévia. A taxa de prematuridade recorrente foi de 42,0%, considerando todas as mulheres com prematuridade prévia. Dentre os 349 prematuros recorrentes, 31,0% foram precoces, 69,0% foram tardios, 62,2% foram espontâneos e 37,8% ocorreram por intervenção obstétrica.

Os prematuros recorrentes, quando comparados aos não recorrentes, foram mais frequentes em mulheres com condições socioeconômicas de classe A/B e C, com três ou mais partos anteriores e com história de natimorto ou óbito neonatal. Dentre os recém-nascidos termo completo, verificou-se maiores proporções de prematuros prévios entre mulheres eutróficas e com sobrepeso, que tiveram três ou mais partos anteriores, cesárea prévia, histórico de natimorto ou óbito neonatal, com síndromes hipertensivas, infecção na admissão para o parto e placenta prévia, em comparação aos recém-nascidos termo completo sem prematuridade prévia ( Tabela 1 ).

**Tabela 1:** Características maternas e de assistência ao parto utilizadas para ponderação, segundo a prematuridade prévia. Brasil, 2011–2012.

	Prematuros (n = 1.215)		p^a^	Termos completos (n = 5.486)		p^a^
	
	Prematuridade prévia			Prematuridade prévia		
	
	Sim	Não		Sim	Não	
Total	349 (100,0)	866 (100,0)		481 (100,0)	5.005 (100,0)	
Tipo de hospital						
Público	190 (54,4)	420 (48,5)	0,172	224 (46,6)	2.157 (43,1)	0,257
Misto	121 (34,7)	338 (39,0)		213 (44,3)	2.305 (46,1)	
Privado	38 (10,9)	108 (12,5)		44 (9,1)	543 (10,8)	
Idade materna^b^						
12 a 19 anos	31 (9,0)	70 (8,1)	0,310	27 (5,6)	313 (6,3)	0,931
20 a 34 anos	257 (74,3)	618 (71,4)		383 (79,6)	3.972 (79,4)	
≥ 35 anos	58 (16,8)	178 (20,6)		71 (14,8)	720 (14,4)	
Classificação socioeconômica^b^						
Classe D/E – baixa	97 (27,9)	222 (25,8)	0,048	130 (27,2)	1.248 (25,2)	0,438
Classe C – média	165 (47,6)	471 (54,9)		251 (52,5)	2.597 (52,3)	
Classe A/B – alta	85 (24,5)	166 (19,3)		97 (20,3)	1.115 (22,5)	
Adequação do pré-natal^b^						
Inadequado ou parcialmente adequado	142 (41,5)	356 (42,5)	0,762	178 (37,6)	1.864 (38,0)	0,925
Adequado ou mais que adequado	200 (58,5)	482 (57,5)		296 (62,4)	3.044 (62,0)	
Fumo no terceiro trimestre da gestação^b^						
Não	319 (91,7)	778 (89,8)	0,490	440 (91,5)	4.572 (91,3)	0,553
Sim, < 10 cigarros/dia	19 (5,5)	51 (5,9)		27 (5,6)	247 (4,9)	
Sim, ≥ 10 cigarros/dia	10 (2,9)	37 (4,3)		14 (2,9)	186 (3,7)	
IMC						
< 18,5	27 (7,7)	52 (6,0)	0,743	23 (4,8)	295 (5,9)	0,011
18,5–24,9	191 (54,7)	484 (55,9)		246 (51,1)	2.845 (56,8)	
25,0–29,9	91 (26,1)	231 (26,7)		150 (31,2)	1.296 (25,9)	
≥ 30,0	40 (11,5)	99 (11,4)		62 (12,9)	569 (11,4)	
Paridade						
1–2 partos anteriores	238 (68,2)	691 (79,8)	0,001	363 (75,3)	4.149 (82,9)	< 0,001
≥ 3 partos anteriores	111 (31,8)	175 (20,2)		118 (24,7)	856 (17,1)	
Cesárea prévia^b^						
Sim	138 (39,5)	328 (38,1)	0,604	232 (48,6)	1.951 (39,2)	< 0,001
Não	211 (60,5)	534 (61,9)		245 (51,4)	3.027 (60,8)	
Natimorto ou óbito neonatal anterior^b^						
Sim	81 (23,2)	65 (7,5)	< 0,001	84 (17,5)	220 (4,4)	< 0,001
Não	268 (76,8)	800 (92,5)		397 (82,5)	4.785 (95,6)	
Malformação^b^						
Sim	13 (3,7)	33 (3,8)	0,944	8 (1,7)	59 (1,2)	0,359
Não	336 (96,3)	833 (96,2)		473 (98,3)	4.946 (98,8)	
Hipertensão crônica^b^						
Sim	27 (7,7)	49 (5,7)	0,176	21 (4,4)	155 (3,1)	0,141
Não	322 (92,3)	817 (94,3)		460 (95,6)	4.850 (96,9)	
Diabetes crônica^b^						
Sim	6 (1,7)	26 (3,0)	0,206	6 (1,2)	64 (1,3)	0,953
Não	343 (98,3)	840 (97,0)		475 (98,8)	4.941 (98,7)	
Complicações clínico-obstétricas						
Síndromes hipertensivas^c^	84 (24,1)	173 (20,0)	0,114	85 (17,7)	472 (9,4)	< 0,001
Diabetes gestacional	38 (10,9)	95 (11,0)	0,967	52 (10,8)	464 (9,3)	0,275
Outra doença crônica severa^d^	6 (1,7)	7 (0,8)	0,163	5 (1,0)	41 (0,8)	0,613
Infecção na admissão para o parto	4 (1,1)	10 (1,2)	0,994	6 (1,2)	9 (0,2)	< 0,001
Descolamento prematuro da placenta	22 (6,3)	40 (4,6)	0,227	8 (1,7)	53 (1,1)	0,229
Placenta prévia	4 (1,1)	19 (2,2)	0,225	6 (1,2)	19 (0,4)	0,007
Crescimento intrauterino restrito	37 (10,6)	78 (9,0)	0,390	20 (4,2)	157 (3,1)	0,226

^a^Teste χ^2^de Rao-Scott.

^b^Total diferente devido a *missing values* .

^c^Hipertensão, pre-eclâmpsia e síndrome HELLP.

^d^Doenças cardíacas crônicas (exceto hipertensão), doenças renais crônicas e doenças autoimunes.

Conforme a Tabela 2 , observa-se que os prematuros recorrentes espontâneos foram mais frequentes em hospitais públicos e em adolescentes, de classe média, com baixo peso e eutróficas, com pré-natal inadequado ou parcialmente adequado, com três ou mais partos anteriores, sem cesárea prévia, com natimorto ou óbito neonatal anterior, malformação, diabetes gestacional, infecção na admissão para o parto e descolamento prematuro da placenta, quando comparados aos recém-nascidos termo completo. Em contrapartida, os prematuros recorrentes por intervenção obstétrica ocorreram mais em mulheres com idade ≥ 35 anos, classe socioeconômica alta, com baixo peso ou obesa, pré-natal adequado ou mais que adequado, com cesariana prévia, natimorto ou óbito neonatal anterior e hipertensão crônica, quando comparados aos recém-nascidos termo completo. Ademais, os prematuros recorrentes por intervenção obstétrica apresentaram a maioria das complicações clínico-obstétricas, exceto doença crônica severa e placenta prévia.

**Tabela 2:** Tipo de prematuridade recorrente segundo características maternas e de assistência ao parto. Brasil, 2011–2012.

	Prematuridade recorrente				39–40 semanas (Ref.) n (%)

	Espontânea^b^	p^a^	Intervenção obstétrica	p^a^	
		
	n (%)		n (%)		
Total	217 (100,0)		132 (100,0)		5.486 (100,0)
Tipo de hospital					
Público	134 (61,8)	< 0,001	57 (43,2)	0,091	2.381 (43,4)
Misto	68 (31,3)		53 (40,2)		2.518 (45,9)
Privado	15 (6,9)		22 (16,7)		587 (10,7)
Idade materna^b^					
12 a 19 anos	28 (13,1)	< 0,001	3 (2,3)	0,007	340 (6,2)
20 a 34 anos	159 (74,3)		98 (74,2)		4.355 (79,4)
≥ 35 anos	27 (12,6)		31 (23,5)		791 (14,4)
Classificação socioeconômica^c^					
Classe D/E – baixa	67 (30,9)	0,163	30 (22,7)	< 0,001	1.378 (25,3)
Classe C – média	113 (52,1)		54 (40,9)		2.848 (52,4)
Classe A/B – alta	37 (17,0)		48 (36,4)		1.212 (22,3)
Adequação do pré-natal^c^					
Inadequado ou parcialmente adequado	106 (50,2)	0,001	37 (28,2)	0,011	2.042 (37,9)
Adequado ou mais que adequado	105 (49,8)		94 (71,8)		3.340 (62,1)
Fumo no terceiro trimestre da gestação					
Não	194 (89,4)	0,488	126 (95,4)	0,291	5.012 (91,3)
Sim, < 10 cigarros/dia	15 (6,9)		5 (3,8)		274 (5,0)
Sim, ≥ 10 cigarros/dia	8 (3,7)		1 (0,8)		200 (3,6)
IMC					
< 18,5	16 (7,4)	0,037	11 (8,3)	0,002	318 (5,8)
18,5–24,9	132 (60,8)		59 (44,7)		3.091 (56,3)
25,0–29,9	57 (26,3)		34 (25,8)		1.446 (26,4)
≥ 30,0	12 (5,5)		28 (21,2)		631 (11,5)
Paridade					
1–2 partos anteriores	139 (64,1)	< 0,001	99 (75,0)	0,084	4.512 (82,2)
≥ 3 partos anteriores	78 (35,9)		33 (25,0)		974(17,8)
Cesárea prévia^c^					
Sim	57 (26,3)	< 0,001	81 (61,4)	< 0,001	2.183 (40,0)
Não	160 (73,7)		51 (38,6)		3.272 (60,0)
Natimorto ou óbito neonatal anterior					
Sim	48 (22,1)	< 0,001	33 (25,0)	< 0,001	304 (5,5)
Não	169 (77,9)		99 (75,0)		5.182 (94,5)
Malformação					
Sim	8 (3,7)	0,020	5 (3,8)	0,058	67 (1,2)
Não	209 (96,3)		127 (96,2)		5.419 (98,8)
Hipertensão crônica					
Sim	3 (1,4)	0,061	24 (18,2)	< 0,001	176 (3,2)
Não	214 (98,6)		108 (81,8)		5.310 (96,8)
Diabetes crônica					
Sim	4 (1,8)	0,694	2 (1,5)	0,995	70 (1,3)
Não	213 (98,2)		130 (98,5)		5.416 (98,7)
Complicações clínico-obstétricas					
Síndromes hipertensivas^d^	17 (7,8)	0,049	67 (50,8)	< 0,001	557 (10,1)
Diabetes gestacional	11 (5,1)	0,019	27 (20,5)	< 0,001	516 (9,4)
Outra doença crônica severa^e^	4 (1,8)	0,123	2 (1,5)	0,430	46 (0,8)
Infecção na admissão para o parto	3 (1,4)	0,030	2 (1,5)	0,056	15 (0,3)
Descolamento prematuro da placenta	8 (3,7)	0,039	14 (10,6)	< 0,001	61 (1,1)
Placenta prévia	2 (0,9)	0,714	2 (1,5)	0,272	25 (0,5)
Crescimento intrauterino restrito	7 (3,2)	0,414	29 (22,0)	< 0,001	177 (3,2)

Ref.: referência

^a^Teste χ^2^de Rao-Scott.

^b^Início espontâneo de trabalho de parto ou ruptura prematura de membranas.

^c^Total diferente devido a *missing values* .

^d^Hipertensão, pré-eclâmpsia e síndrome HELLP.

^e^Doenças cardíacas crônicas (exceto hipertensão), doenças renais crônicas e doenças autoimunes.

A análise múltipla mostrou maiores chances de prematuridade recorrente espontânea em adolescentes, de classe baixa e que fumavam 10 ou mais cigarros por dia no terceiro trimestre de gestação. Por outro lado, mulheres com idade materna ≥ 35 anos, de classe socioeconômica alta, com cesárea prévia, hipertensão crônica e diabetes crônica apresentaram maiores chances de prematuridade recorrente por intervenção obstétrica, quando comparados aos recém-nascidos termo completo ( Tabela 3 ).

**Tabela 3:** Características maternasa utilizadas para ponderação das mulheres, segundo o tipo de prematuridade recorrente. Brasil, 2011–2012.

	Prematuridade recorrente espontânea^b^(n = 197)	p^c^	Prematuridade recorrente por intervenção obstétrica^b^(n = 136)	p^c^
	
	Crude OR (IC95%)		Crude OR (IC95%)	
Idade materna				
12 a 19 anos	2,08 (1,60–2,71)	< 0,001	0,76 (0,47–1,24)	0,991
20 a 34 anos	1,00		1,00	
≥ 35 anos	1,02 (0,81–1,27)	0,912	1,74 (1,39–2,19)	< 0,001
Classificação socioeconômica				
Classe D/E – baixa	1,00		1,00	
Classe C – média	0,73 (0,61–0,88)	< 0,001	1,29 (1,00–1,68)	0,063
Classe A/B – alta	0,51 (0,40–0,64)	< 0,001	1,89 (1,43–2,51)	< 0,001
Fumo no terceiro trimestre da gestação				
Não	1,00		-	
Sim, < 10 cigarros/dia	1,36 (0,98–1,90)	0,070	-	-
Sim, ≥ 10 cigarros/dia	1,74 (1,22–2,47)	0,002	-	-
Paridade				
1–2 partos anteriores	1,00		1,00	
≥ 3 partos anteriores	1,43 (1,18–1,73)	< 0,001	1,32 (1,04–1,67)	0,023
Cesárea prévia				
Não	-	-	1,00	
Sim	-	-	2,30 (1,89–2,81)	< 0,001
Natimorto ou óbito neonatal anterior				
Não	1,00		1,00	
Sim	2,21 (1,70–2,87)	< 0,001	2,82 (2,11–3,76)	< 0,001
Hipertensão crônica				
Não	1,00		1,00	
Sim	1,01 (0,63–1,59)	0,990	3,06 (2,12–4,40)	< 0,001
Diabetes crônica				
Não	1,00		1,00	
Sim	1,31 (0,67–2,57)	0,447	3,09 (1,74–5,49)	< 0,001

*OR: odds ratio;* IC95%: intervalo de confiança de 95%.

^a^Todas as variáveis foram selecionas baseando-se no DAG (gráfico aciclíco direcionado).

^b^Diferenças devido a *missing value* .

^c^Os desfechos foram comparados com a categoria: 39–40 semanas de gestação.

A Tabela 4 mostra os balanceamentos antes e após a realização do escore de propensão, por meio das diferenças padronizadas entre as médias dos grupos. Antes do balanceamento, verificou-se que a maior diferença de média foi para natimorto ou óbito neonatal anterior (0,422) para ambos os grupos. Após a ponderação, verificou-se que as diferenças padronizadas entre as médias dos dois grupos aproximaram-se de zero para todas as covariáveis, indicando que o balanceamento após o ajuste pelo escore de propensão foi adequado.

**Tabela 4:** Diferença de médias para as características utilizadas na ponderação das mulheres, segundo o tipo de prematuridade recorrente. Brasil, 2011–2012.

	Prematuridade recorrente espontânea		Prematuridade recorrente por intervenção obstétrica	
	
	Antes de balancear	Após balancear	Antes de balancear	Após balancear
Idade materna				
12 a 19 anos	0,034	-0,026	0,034	-0,026
20 a 34 anos	-0,021	0,009	-0,021	0,009
≥ 35 anos	-0,000	0,026	-0,000	0,026
Classificação socioeconômica				
Classe D/E - baixa	0,044	0,050	0,044	0,050
Classe C - média	-0,018	-0,049	-0,018	-0,049
Classe A/B - alta	-0,025	0,004	-0,025	0,004
Fumo no terceiro trimestre da gestação				
Não	-0,015	0,035	-	-
Sim, < 10 cigarros/dia	0,042	-0,027	-	-
Sim, ≥ 10 cigarros/dia	-0,031	-0,021	-	-
Paridade				
1–2 partos anteriores	-0,222	-0,056	-0,222	-0,056
≥ 3 partos anteriores	0,222	0,056	0,222	0,056
Cesárea prévia				
Sim	-	-	0,089	-0,018
Não	-	-	-0,089	0,018
Natimorto ou óbito neonatal anterior				
Sim	0,422	-0,035	0,422	-0,035
Não	-0,422	0,035	-0,422	0,035
Hipertensão crônica				
Sim	0,099	0,002	0,099	0,002
Não	-0,099	-0,002	-0,099	-0,002
Diabetes crônica				
Sim	0,005	-0,003	0,005	-0,003
Não	-0,005	0,003	-0,005	0,003

Diante disso, a análise final mostrou que as mulheres com prematuridade prévia têm 3,89 vezes a chance de terem prematuridade recorrente espontânea (ORaj: 3,89; IC95% 3,01–5,03) e 3,47 vezes a chance de terem prematuridade recorrente por intervenção obstétrica (ORaj: 3,47; IC95% 2,59–4,66), quando comparadas às mulheres com recém-nascidos termo completo ( Tabela 5 ).

**Tabela 5:** Razões de chances brutas e ajustadas ao comparar prematuros recorrentes com recém-nascidos termo completo, após o escore de propensão. Brasil, 2011–2012.

	OR bruta (IC95%)	OR após balanceamento (IC95%)
Prematuridade recorrente		
Geral		
< 37 semanas	4,25 (3,62–4,97)	3,72 (3,01–4,61)
39–40 semanas	1,00	
Espontânea		
< 37 semanas	4,10 (3,39–4,95)	3,89 (3,01–5,03)
39–40 semanas	1,00	1,00
Intervenção obstétrica		
< 37 semanas	4,48 (3,59–5,60)	3,47 (2,59–4,66)
39–40 semanas	1,00	1,00

*OR: odds ratio;* IC95%: intervalo de confiança de 95%.

## DISCUSSÃO

A taxa de prematuridade recorrente foi de 42,0% dentre as mulheres com prematuridade prévia, dos quais a maioria foi tardio e por causa espontânea. Fatores relacionados à vulnerabilidade social apresentaram maiores chances para a prematuridade recorrente espontânea, ao passo que melhores condições socioeconômicas associaram-se à prematuridade recorrente por intervenção obstétrica. Além disso, a prematuridade prévia aumentou as chances de recorrência da prematuridade espontânea e por intervenção obstétrica.

A taxa de recorrência em nosso estudo foi maior do que as relatadas em estudos realizados em Holanda (29,3%)^22^ , Japão (22,3%)^23^ e Utah (21,0%)^12^ . As razões para isso ainda são pouco conhecidas, todavia, estudos mostram que fatores socioeconômicos, assistência pré-natal inadequada, comportamentos maternos de risco, complicações obstétricas, fatores genéticos e modelos de assistência obstétrica são possíveis fatores determinantes para a prematuridade recorrente^4 , 12 , 24^ .

Ao analisar a prematuridade recorrente pelo tipo de parto, verificaram-se maiores frequências de prematuros espontâneos (62,2%). Além disso, adolescentes com piores condições socioeconômicas apresentaram maiores chances de terem prematuridade recorrente espontânea, ao passo que mulheres com melhores condições socioeconômicas, cesárea prévia, hipertensão crônica e diabetes crônica foram significativamente associadas à prematuridade recorrente por intervenção obstétrica. Esses achados corroboram com estudos prévios brasileiros que identificaram que mulheres em situações de vulnerabilidade social têm maiores riscos de prematuros espontâneos, enquanto aquelas com melhores condições socioeconômicas apresentam maiores riscos de prematuros por intervenção obstétrica^25 , 26^ . Ademais, observou-se valores significativamente mais altos de prematuros em mulheres com baixo peso ou obesas. É reconhecido que a nutrição inadequada está intimamente relacionada ao baixo nível socioeconômico das gestantes, assim como o excesso de peso está associado às intercorrências maternas (diabetes gestacional e síndromes hipertensivas). Logo, o ganho de peso gestacional diferente do recomendado leva a maiores riscos de desfechos adversos para mães e seus recém-nascidos^27 , 28^ .

Este estudo também revelou maiores chances de recorrência de parto prematuro espontâneo e por intervenção obstétrica independentemente do tipo de prematuridade prévia. Coorte retrospectiva realizada em 20 hospitais localizados em Utah mostrou que a prematuridade prévia espontânea é um forte preditor de prematuridade espontânea subsequente (RRaj: 5,64; IC95% 5,27–6,05), assim como a prematuridade prévia por intervenção obstétrica apresenta maiores riscos de prematuridade recorrente por intervenção médica (RRaj: 9,10; IC95% 4,68–17,71), e vice-versa^29^ .

No Brasil, é possível que mulheres com prematuridade prévia por intervenção obstétrica tenham riscos ainda mais elevados de recorrência, em virtude dos efeitos da organização da assistência obstétrica e da escolha da mulher pelo mesmo tipo de parto, especialmente a cesariana. Domingues et al.^30^ mostraram que multíparas com cesariana prévia têm preferência inicial pela cesariana em gestações subsequentes. Dentre os motivos para essa escolha, o estudo aponta a possibilidade de agendamento da cesariana ainda no início da gestação^30^ . Em consequência disso, estudo de Nakamura-Pereira et al.^31^ , utilizando a Classificação de Robson, evidenciou que multíparas com cesárea prévia e apresentação cefálica ≥ 37 semanas representa o segundo grupo que mais contribui com as taxas de cesarianas no Brasil. Outro estudo de Nakamura-Pereira et al.^32^ também identificou que dentre as mulheres elegíveis para tentarem o trabalho de parto após uma cesariana, 66,1% tiveram cesariana eletiva de repetição, o que demonstra a adesão ao ditado “uma vez cesárea, sempre cesárea”. Esses fenômenos estão intrinsecamente relacionados com o aumento de partos cada vez mais precoces, que contribuem com quase 10% das taxas de cesariana no Brasil^31^ .

Além da cesariana eletiva, as complicações clínicas maternas também se relacionam com a prematuridade por intervenção obstétrica. Coorte retrospectiva realizada na região Norte da Tanzânia mostrou que mulheres que tiveram pré-eclâmpsia em gestações anteriores apresentaram risco 50% maior de prematuridade recorrente em comparação às mulheres com pressão arterial normal^33^ . Por isso, recomenda-se que mulheres com histórico de prematuridade associada a comorbidades sejam identificadas precocemente e oportunamente tratadas no período pré-natal e durante o trabalho parto para prevenir desfechos materno-fetais negativos.

A recorrência de prematuridade também é influenciada pelo número de prematuros anteriores, ordem de nascimento e idade gestacional^2 , 29 , 34^ . Em coorte de mulheres com três gestações únicas consecutivas, Hiersch et al.^2^ encontraram RR = 3,1 (IC95% 1,9–4,9) para prematuridade recorrente na terceira gestação em mulheres que tiveram prematuro somente na primeira gestação; RR = 5,6 (IC95% 3,6–8,8) em mulheres que tiveram esse desfecho na segunda gestação; e RR = 38,2 (IC95% 20,6–70,8) em mulheres com prematuridade nos dois primeiros partos, quando comparadas às mulheres que tiveram recém-nascido a termo. Portanto, a recorrência em uma terceira gestação está mais associada às mulheres com histórico de parto prematuro na segunda gravidez do que na primeira^34^ . Quanto à idade gestacional, coorte retrospectiva realizada na Califórnia evidenciou que mulheres com primeiro parto antes de 32 semanas de gestação apresentaram risco 23,3 vezes maior de recorrência antes de 32 semanas de gestação^35^ , logo, quanto mais precoce for o nascimento anterior, maior será o risco de nascimento prematuro recorrente.

Quanto às intervenções para prevenir nascimentos cada vez mais precoces, Mazaki-Tovi et al.^9^ em revisão de literatura afirmam que a melhor estratégia ainda é administração de progesterona. A cerclagem uterina também é possível, mas somente na presença de insuficiência cervical uterina, ou em mulheres com história prévia de insuficiência cervical, ou ainda em mulheres com encurtamento cervical uterino precoce diagnosticado por ultrassonografia^9 , 36^ .

O ponto de maior destaque deste estudo foi estimar a chance de prematuridade recorrente em multíparas do Brasil a partir do inquérito nacional “Nascer no Brasil”, que utilizou uma amostra representativa de mulheres considerando as regiões do país, localização geográfica (capital ou interior) e o tipo de atendimento hospitalar (privado, público ou misto). Também se sobressaiu o método de análise aplicado – ponderação pelo escore de propensão –, que permitiu aproximar os resultados deste estudo aos de um estudo experimental, tornando os grupos comparáveis e os resultados mais robustos.

Contudo, este estudo apresenta algumas limitações. Foram incluídas apenas puérperas atendidas em hospitais com mais de 500 partos/ano (que representam 80% dos nascimentos ocorridos no país) e, portanto, é possível que mulheres com partos em hospitais menores ou com partos domiciliares ou em via pública, tenham riscos diferentes para prematuridade recorrente. Também não foi possível estimar o efeito direto do tipo de prematuridade prévia sobre o tipo de prematuridade recorrente, devido à ausência de informação sobre as gestações anteriores. Ademais, não foi possível analisar a prematuridade segundo a idade gestacional em virtude das baixas frequências de recém-nascidos em cada subgrupo de prematuridade recorrente. Estudos futuros deverão incluir esses fatores para uma investigação completa dos riscos para prematuridade recorrente espontânea e por intervenção obstétrica.

Em conclusão, a prematuridade prévia revelou-se um forte preditor para recorrência de prematuridade espontânea e por intervenção obstétrica. Infelizmente, o Brasil situa-se entre os dez países que juntos contribuem com 60% dos nascimentos prematuros do mundo^37^ . Além de trazer implicações para a saúde da criança, a prematuridade também representa a principal causa de morte neonatal e, por isso, o Brasil enfrenta o grande desafio de reduzir as suas taxas de prematuridade. Dessa forma, os achados deste estudo têm implicações clínicas importantes para o monitoramento e manejo de gestantes com histórico de prematuridade, os quais visam auxiliar os profissionais de saúde a realizarem um planejamento com cuidados adequados para prevenir novos partos prematuros e, consequentemente, reduzir os riscos de desfechos neonatais adversos nessa população.

## Footnotes

a Disponíveis em: Dias BAS. Recorrência da cesariana e da prematuridade na pesquisa Nascer no Brasil [tese]. Rio de Janeiro: Escola Nacional de Saúde Pública Sergio Arouca, Fundação Oswaldo Cruz; 2021 [citado 6 jan 2022]. Disponível em: https://www.arca.fiocruz.br/bitstream/icict/49461/2/barbara_almeida_soares_dias_ensp_dout_2021.pdf
